# Glucagon-Like Peptide-2 Modulates Enteric Paneth Cells Immune Response and Alleviates Gut Inflammation During Intravenous Fluid Infusion in Mice With a Central Catheter

**DOI:** 10.3389/fnut.2021.688715

**Published:** 2021-09-03

**Authors:** Guifang Deng, Qiucheng Lei, Xuejin Gao, Yupeng Zhang, Huazhen Zheng, Jingcheng Bi, Xinying Wang

**Affiliations:** ^1^Research Institute of General Surgery, Affiliated Jinling Hospital, Medical School of Nanjing University, Nanjing, China; ^2^Department of Clinical Nutrition, Union Shenzhen Hospital of Huazhong University of Science and Technology, Shenzhen, China; ^3^Department of Hepatopancreatic Surgery, The First People's Hospital of Foshan, Foshan, China; ^4^Department of Clinical Laboratory, The First People's Hospital of Foshan, Foshan, China; ^5^Department of General Surgery, Taizhou People's Hospital, Taizhou, China

**Keywords:** glucagon-like peptide-2, parenteral nutrition, inflammation, Paneth cells, intestinal immunity, intravenous infusion

## Abstract

**Background:** Glucagon-like peptide-2 (GLP-2) has protective effects on gastrointestinal functions. Our previous study found that GLP-2 could significantly reduce intestinal permeability and bacterial translocation in total parenteral nutrition (TPN) animal model. However, the effects of GLP-2 on the impairment of the intestinal Paneth cells immune function and gut inflammation during intravenous fluid infusion mainly consisted of nutritional materials is currently scattered.

**Objective:** The current study was aimed to investigate the efficacy of the GLP-2 in alleviating gut inflammation and modulating enteric Paneth cells immune response in parenterally fed mice and its underlying mechanisms.

**Methods:** Thirty-six male ICR mice underwent venous catheterization were divided into 3 groups: Chow, TPN, and TPN+GLP-2 groups. GLP-2 was administered intravenously at 60 μg/day for 5 days. The small intestine tissue and serum samples were collected on the 7th day.

**Results:** Compared with the TPN group, the expression of tight junction proteins occludin and claudin-1 were significantly increased in the TPN+GLP-2 group. In addition, the expression of lysozyme, sPLA2, insulin-like growth factor-1, and epithelial protection and repair genes were improved in the TPN+GLP-2 group. The levels of IL-6 and TNF-α proteins and mRNAs in the ileum tissues were remarkably reduced in the TPN+GLP-2 group, while IL-10 protein and mRNA level were elevated in the TPN+GLP-2 group (all *p* < 0.05). Moreover, the TPN+GLP-2 group has higher levels of serum endotoxin, D-lactic acid, and MPO than those of the TPN group.

**Conclusions:** GLP-2 alleviated gut inflammation and improved enteric Paneth cells immune responses through intravenous fluid infusion, possibly by improving the functioning of epithelial protection and repair, and reducing mucosal inflammatory responses.

## Introduction

Intravenous (IV) infusion is a method that uses the principle of atmospheric pressure and hydrostatic pressure to input a large number of sterile liquids, electrolytes and drugs into the body through the vein. Due to different injection sites, it can be divided into peripheral venous infusion and central venous infusion. IV infusion is an indispensable method in parenteral nutritional therapy, through which the nutrients needed of patients are supplied. Parenteral nutrition (PN) has been the standardized treatment of patients with acute and/or chronic intestinal failure (1, 2). Some diseases make patients unable to tolerate enteral nutrition and can only rely on parenteral nutrition support, such as short bowel syndrome, small bowel resection, Crohn's disease or intestinal ischemia related to thrombosis, volvulus and trauma, intestinal obstruction and radiation enteritis (3). TPN is also suitable for patients with malnutrition during critical illness (4). Reported, nearly 400,000 patients in the United States rely on parenteral nutrition to survive every year, including more than 1,000 infants with congenital intestinal atresia (5, 6). Despite the apparent benefits, patients receiving PN via IV infusion are more likely to develop severe or fatal complications such as liver injury, intestinal atrophy, and immune function suppression (1, 2). Other impairments that may occur during PN via IV infusion include damage to the intestinal mucosal barrier, intestinal inflammatory response, increased cytokine release, and gut bacterial translocation (2).

Glucagon-like peptide-2 (GLP-2) is a 33-amino-acid proglucagon-derived peptide (7). It is produced by the enteroendocrine L cells in the intestinal epithelium, which is primarily located in the terminal ileum and proximal colon (7). Some research indicated that GLP-2 can promote crypt cell proliferation and mucosal healing, increase intestinal blood flow, improve gut barrier function, and exert an anti-inflammatory effect (7–9). It has shown good therapeutic potential in experimental models after receiving total parenteral nutrition after partial bowel resection. In our previous study, we found that GLP-2 could significantly reduce intestinal permeability and bacterial translocation during parenteral nutrition (10), exhibiting its potential protective role on the intestinal barrier in total parenteral nutrition (TPN). Although some studies have provided insights into the protective effects of GLP-2 on short bowel syndrome and bowel injury (11–13), the available information regarding the effects of GLP-2 on the impairment of the intestinal Paneth cells immune function and gut inflammation during TPN is currently scattered.

Therefore, we investigate the protective effects of GLP-2 against intestinal immune function damage and gut inflammation during intravenous fluid infusion via a central catheter and its possible mechanisms.

## Materials and Methods

### Animals

This study was approved by the animal ethics committee of Jinling hospital (Ethical approval number: 2018NZGKJ-034). A total of 36 male (aged 6–8 weeks) ICR mice were provided by the experimental animal research center of Jinling hospital. The animals initially weighed 20–25 g and were housed 12:12h light/dark cycle under suitable humidity and temperature conditions. Mice were freely accessed to water and food and with their activity unrestricted prior to the study (14). The mice were allowed to acclimatize to laboratory conditions for one week before the experimental protocol was initiated.

### Study Design

Thirty-six male ICR mice were randomly divided into three groups: Chow (Control group, *n* = 12), TPN (TPN + vehicle group, *n* = 12), and TPN + GLP-2 (TPN + GLP-2 group, *n* = 12). The Control group mice were fed with standard laboratory chow diet, while the other two experimental groups were respectively treated with 24 h continuous pumping of parenteral nutrition solution or parenteral nutrition combined with GLP-2 analog (teduglutide, PLLabs, Canada). GLP-2 therapy was provided intravenously at a dose of 60 μg/day (30 μg intravenously, twice daily). This dose was selected because, in our previous studies, we found a positive effect of GLP-2 during TPN at the same dose (10).

### Animal Experiments

The animal experiments were performed at a temperature of 24°C and a humidity of 40–50%. Mice were anesthetized by intraperitoneal ketamine injection (100 mg/kg) and venous catheter (Helix Medical Inc., Carpentaria, CA, USA) was surgically implanted in their right internal jugular vein, after which they were adapted to diet treatment for 24 h post-surgery (10). During the 48 h recovery period after surgery, the mice were treated with 0.9% saline through the catheter. Then mice received parenteral nutrition solution at rates of 4.4 mL/day 1, 7.7 mL/day 2, and 11 mL/ day 3–5 in the TPN and TPN+GLP-2 groups, as previously described (10). The Chow group mice were continuous intravenous pumping of saline via the right internal jugular vein, and had free access to water and Chow diet. Dissolve GLP-2 in sterile PBS and mice were intravenous injection 30 μg of GLP-2 (a total of 100 μL of solution) twice daily for five days in the TPN+GLP-2 group. Mice in the Control and TPN group were also injected with the vehicle (100 μL of PBS) without GLP-2. The TPN solution provided approximately 1,951 kJ/kg per day of energy and 16.4 g/kg per day of protein. The PN formula mainly includes the following: 5.3% free amino acids, 2.1% fat emulsion, 32% dextrose, multivitamins and electrolytes, with total calories 1,280 kcal/L, and the non-protein calories and nitrogen ratio 149:1 (15). These values could meet the nutritional and energetic needs of mice weighing 25–30 g (10). In order to ensure that all three groups of mice receiving the same quantity of calories and proteins, each mouse is raised in a single cage. The control group calculated the amount of standard laboratory feeds every day.

Three groups of mice were anesthetized with 1% pentobarbital sodium (30 mg/ kg) and then sacrificed after feeding for 5 days (i.e., 8 days after catheterization). We obtained portal vein blood samples and small intestine tissue. All the intestine tissue and serum samples were stored in the refrigerator at −80°C for later testing.

### Transmission Electron Microscopy (TEM)

A segment of the small intestine tissues approximately 0.5 cm in length from each sample was processed for examination under TEM.

### Immunofluorescence for Detection of Zonula Occludens-1 (ZO-1), Occludin, and Claudin-1 Tight Junction Proteins in the Ileum Tissues

The fixed ileum tissues were processed (Tissue-Tek VIP; Sakura Finetek Japan, Tokyo, Japan) and embedded in paraffin. The tissue sections were cut to a thickness of 5 μm and deparaffinized. Antigen retrieval was performed by immersing sections in sodium citrate buffer (pH = 6.0) at 95°C for 30 min. The tissue sections were blocked in blocking buffer (5% normal goat serum, 5% bovine serum albumin, and 0.1% Tween 20 in sterile PBS) at 20–24° Croom temperature, and then incubated with primary antibody at 4°C. The tissue sections were washed three times in sterile PBS and incubated with secondary antibody at 20–24° Croom temperature for 60 min on the following day. Subsequently, after 3 washes with sterile PBS, the tissue sections were mounted in 50% glycerol, photographed, and observed under a confocal microscope (Olympus, Tokyo, Japan). The evaluation procedure of acquired images were conducted blindly with the Image Pro Plus software. Rabbit anti-mouse occludin, claudin-1, and ZO-1 antibodies (Abcam, Cambridge, UK) at dilutions of 1:200 were then used for immunofluorescence (IF).

### Western Blotting

The total concentrations of proteins in small intestinal tissues were determined using the bicinchoninic acid protein assay kit (Sangon Biotech Co., Shanghai, China). The protein concentration of each sample was 4 μg/μL. The total protein (50 μg) was electrophoresed on 8 or 10% sodium dodecyl sulfate polyacrylamide gel. The separated proteins were transferred to a PVDF membrane (Millipore Co., Billerica, MA, USA). Block the membrane for non-specific binding proteins in 5% skimmed milk at room temperature 20–24°C for 1 h, and then incubated overnight at 4°C with Anti-Lysozyme antibody (1:1,000; Abcam, Cambridge, MA, UK), Anti-occludin (1:1,000; Abcam, Cambridge, MA, UK), Anti-claudin-1(1:1,000; Abcam, Cambridge, MA, UK), Anti-secretory phospholipase A2 antibody rabbit (1:1,000; Abcam, Cambridge, MA, UK), and Anti-GAPDH antibody (1:10,000; Santa Cruz Biotechnology, MA, USA). Subsequently, the membrane was washed three or five times in Tris-buffered saline containing Tween-20 and incubated with appropriate species-specific secondary antibodies at 20–24°C for 1 h at room temperature. Use chemiluminescence detection reagents and expose them to Kodak XAR film. Using the GAPDH bands as an internal control, all band densities were analyzed with ImageJ software.

### Quantitative Real-Time PCR

The expression levels of IL-6, IL-10, TNF-α, trefoil factor-1,2 and 3 (Tff-1,Tff-2, and Tff-3), transforming growth factor-b1, 2 and 3(Tgfb-1,Tgfb-2, and Tgfb-3), epidermal growth factor receptor (Egfr), hepatocyte growth factor (Hgf), keratinocyte growth factor (Fgf7), and insulin like growth factor-1(Igf1) mRNA in the terminal ileum tissue were analyzed by qRT-PCR. The primers for gene sequences were provided by GenScript Co., Ltd. (Nanjing, China), and are listed in Supplementary Table 1. Use TRIzol reagent (TakaRa, Dalian, China) to extract total RNA from ileal tissue according to the instructions. Prime-Script RT kit (TaKaRa, Dalian, China) was used to reverse transcribed RNA into cDNA, according to the instructions. The 2^−ΔΔCt^ method was used.

### Enzyme-Linked Immunosorbent Assay

ELISA kits for mouse TNF-α, mouse IL-6, mouse IL-10 (Nanjing BioSky Sci & Tech Co., Ltd., Nanjing, China) and mouse insulin like growth factor-1 (Abcam, Cambridge, MA, UK) were used to determine the levels of TNF-α, IL-6, IL-10, and Igf-1 proteins, respectively, in small intestinal tissue homogenates, according to the manufacturer's instructions.

A mouse D-lactate ELISA kit (R&D Systems Inc., Minneapolis, MN, USA) was also used to determine serum D-lactate levels, while following the manufacturer's instructions.

### Intestinal Tissue Myeloperoxidase (MPO) Activity Assay

An MPO Activity Assay Kit (R&D Systems Inc., Minneapolis, MN, USA) was used to determine the MPO levels in small intestinal tissue homogenates, while following the manufacturer's instructions.

### Serum Endotoxin Activity Assay

Serum endotoxin levels were determined using an Endotoxin Activity Assay Kit (EAATM, Spectral Diagnostics Inc., Xiamen, China), according to the manufacturer's instructions.

### Statistical Analyses

Data were expressed as mean ± standard deviation values for each treatment group. The results were compared between all three treatment groups by one-way analysis of variance (ANOVA) and then the Fisher's test was used for *post-hoc* analysis. If there were heterogeneous variances among groups, Dunnett's T3 test was used instead. A *P* < 0.05 was considered statistically significant. And using SPSS 21.0 software (SPSS Inc., Chicago, IL, USA) for analysis.

## Results

### Effects of GLP-2 on Intestinal Tissue Tight Junctions and Tight Junction Proteins

**Figure 1:**
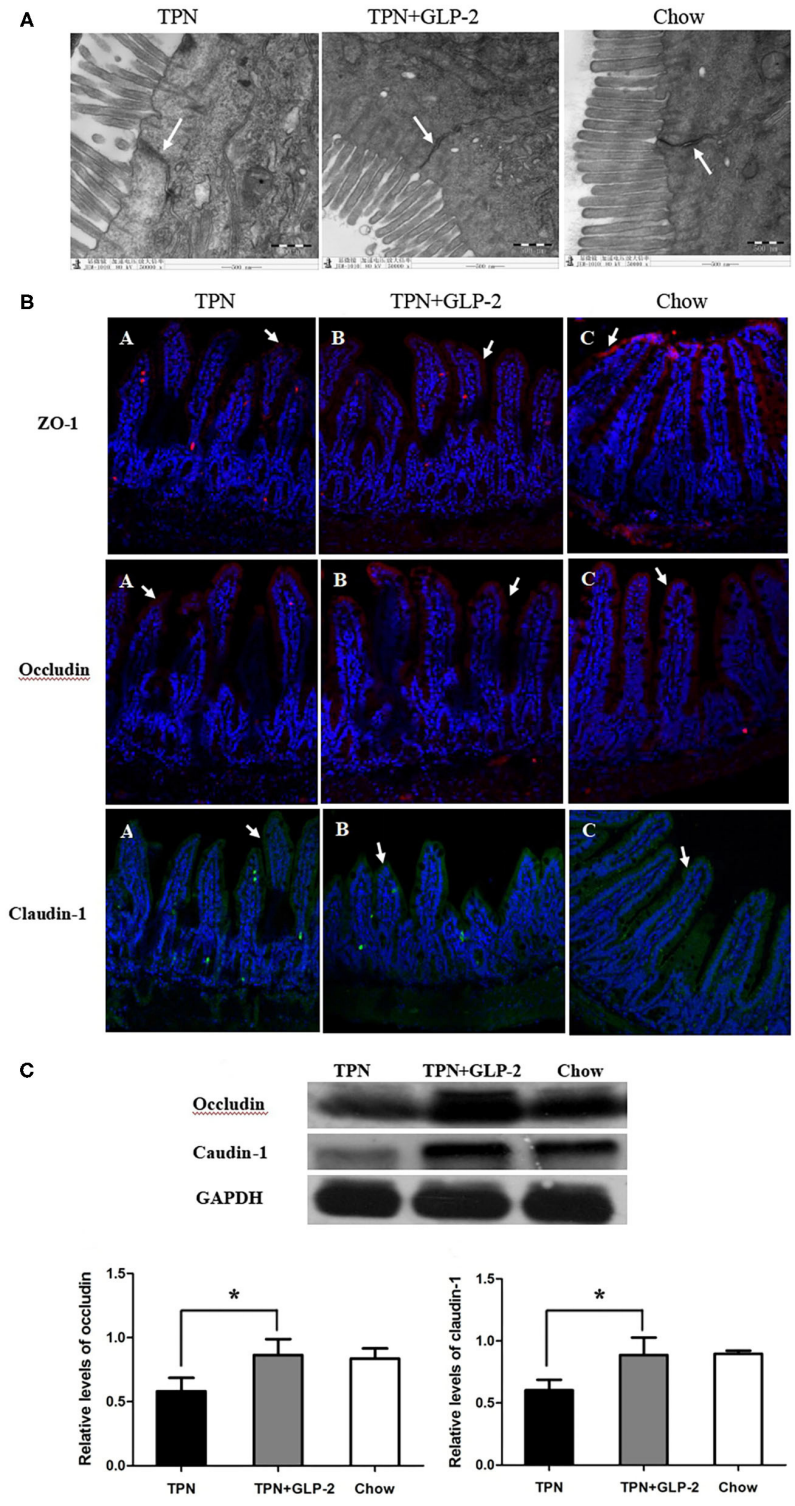
The tight junctions and tight unction proteins. **(A)** Transmission electron microscopy. **(B)** Immunofluorescence. **(C)** Western blot analysis. Where ^*^indicates *P* < 0.05 for TNP vs. TPN+GLP-2 and Chow.

TEM demonstrated that the surface of the intestinal epithelial cell membrane was normal, the tight junction structure between cells completed, the bridge density high, and the connections dense in the Chow and TPN+GLP-2 groups. The surface of the intestinal epithelial cell membrane was reduced, absent, or lodging, the tight junction structure between cells less complete and partially broken, the bridge particle density decreased, the connections loose, and the gaps between cells widened in the TPN group (Figure 1A).

Immunofluorescence showed that the tight junction proteins ZO-1, occludin, and claudin-1 were located on the surfaces of the villi, and their distributions were continuous and dense in the Chow and TPN+GLP-2 groups. The distributions of the fluorescence of the tight junction proteins ZO-1, occludin, and claudin-1 were discontinuous, partially fragmented, partially displaced into cell interiors, and low in density in the TPN group (Figure 1B).

Compared with the Chow and TPN+GLP-2 groups, Western blot analysis showed that the levels of occludin and claudin-1 proteins in intestinal tissues were significantly decreased in the TPN group (*P* < 0.05) (Figure 1C).

### Effects of GLP-2 on Paneth Cells Function in Intestinal Tissue

**Figure 2:**
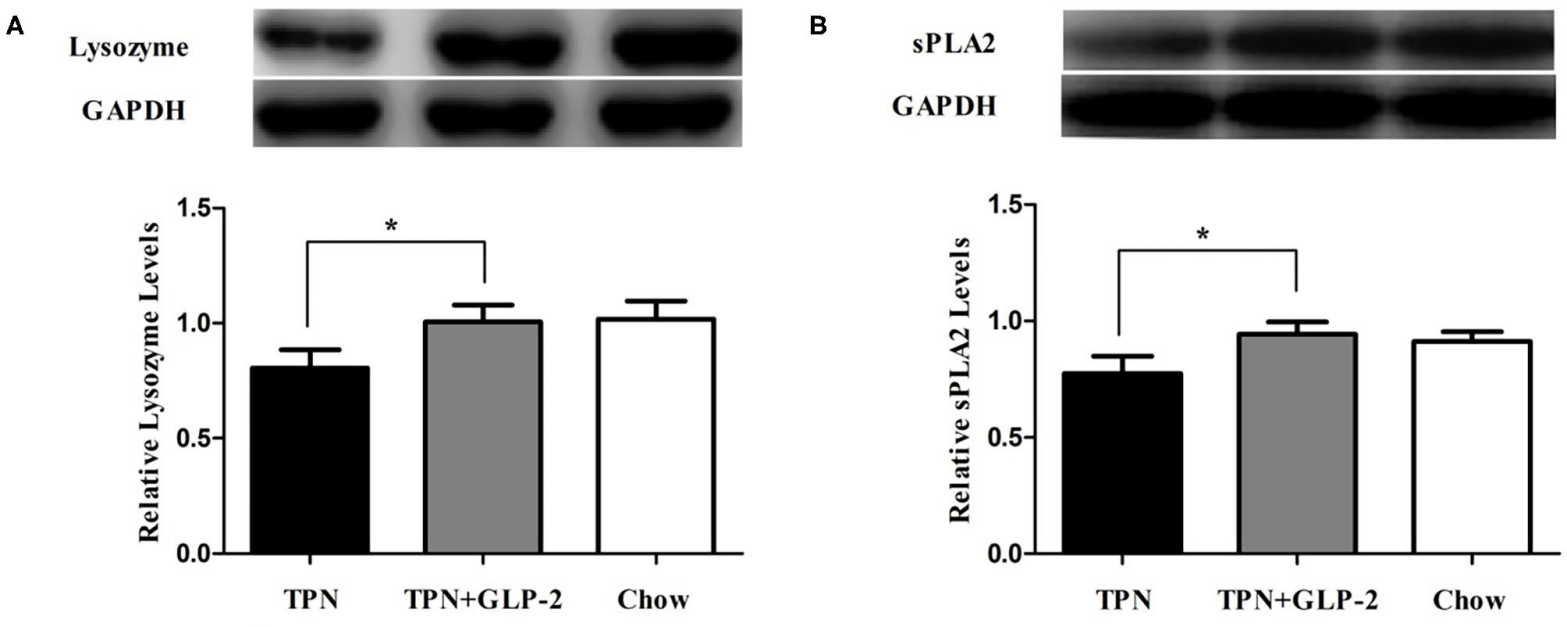
The intestinal tissue Paneth cells function. **(A)** Lysozyme. **(B)** Secreted phospholipase A2. Where ^*^indicates *p* < 0.05 for TNP vs. TPN+GLP-2 and Chow.

To detect the effects of GLP-2 on Paneth cells function in intestinal tissue, we measured the expression of Lysozyme and secretory phospholipase A2 proteins (sPLA2) in intestinal tissue. Compared with the TPN+GLP-2 group, the levels of Lysozyme and sPLA2 were significantly decreased in the TPN group (*P* < 0.05), and no significant difference between the remaining two groups has observed (Figures 2A,B).

### Effect of GLP-2 on Intestinal Epithelial Protection and Repair in Intestinal Tissue

**Figure 3:**
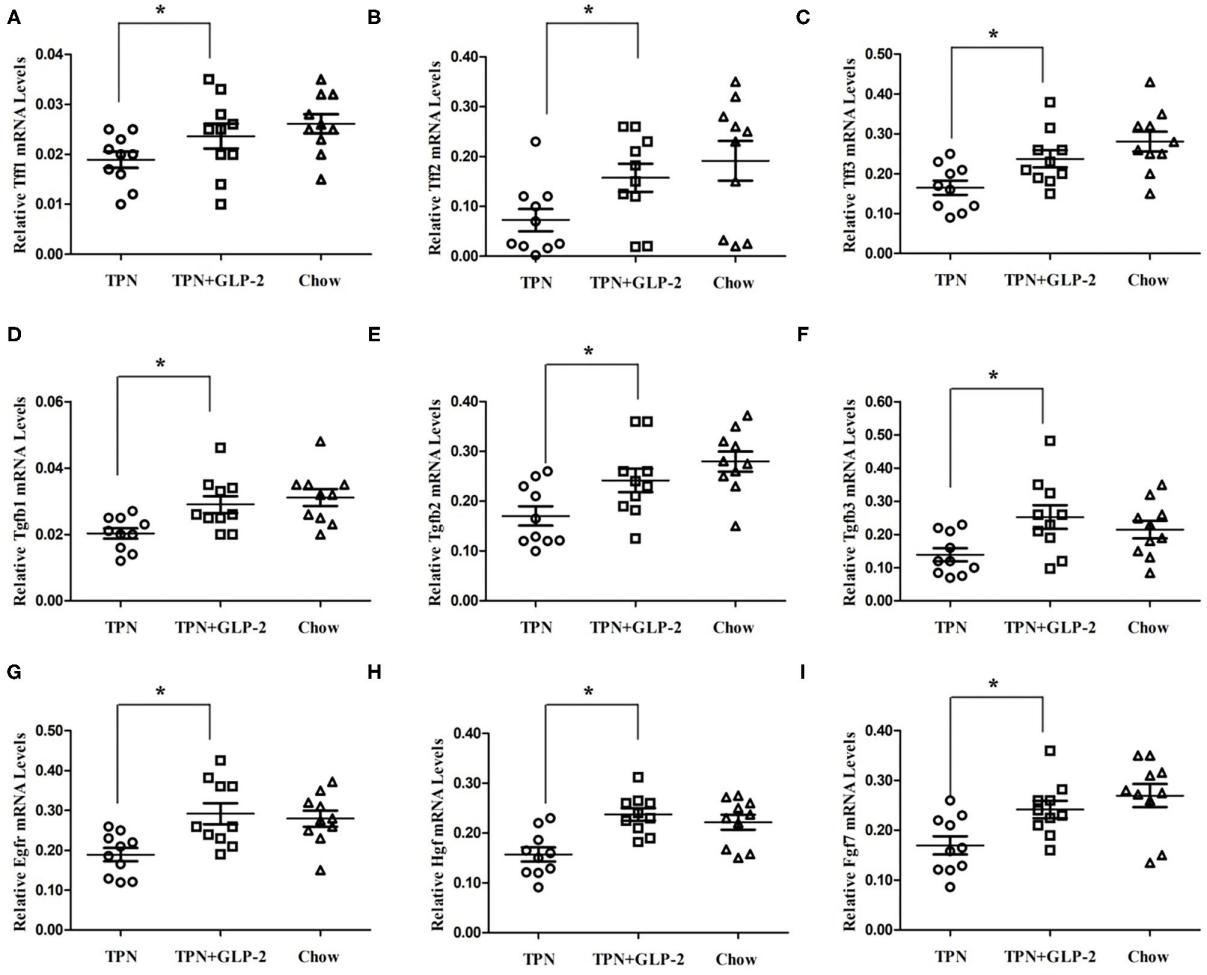
The intestinal tissue the expression of epithelial protection and repair genes. **(A–I)** [trefoil factor-(Tff)1,2 and 3; transforming growth factor-(Tgf) b1,2 and 3; epidermal growth factor receptor (Egfr); hepatocyte growth factor (Hgf); keratinocyte growth factor (Fgf7)], where ^*^indicates *p* < 0.05 for TNP vs. TPN+GLP-2 and Chow.

Then, we detected the mRNA levels of *Tff1, Tff2, Tff3, Tgfb1, Tgfb2, Tgfb3, Egfr, Hgr* and *Fgf7*, genes of epithelial protection and repair. Compared with the TPN group, the expression of all above genes were increased in the TPN+GLP-2 group (*P* < 0.05), and no difference between the TPN+GLP-2 and Chow groups (Figures 3A–I).

### Effects of GLP-2 on the Expression of Insulin Like Growth Factor-1 in Intestinal Tissue

**Figure 4:**
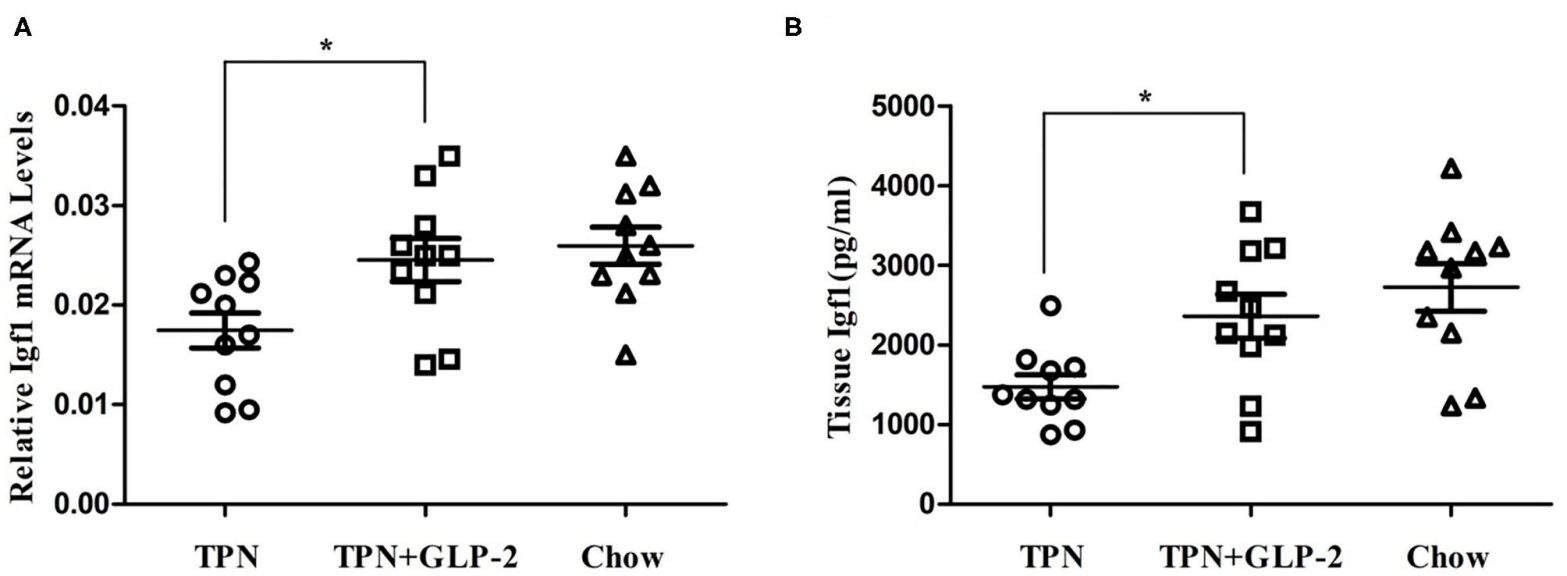
The intestinal tissue the expression of insulin like growth factor-1. **(A)** Expression of insulin like growth factor-1 mRNA. **(B)** Expression of insulin like growth factor-1 protein. Where ^*^indicates *p* < 0.05 for TNP vs. TPN+GLP-2 and Chow.

We also measured the level of IGF-1. Mice in the TPN+GLP-2 group have higher mRNA level of IGF-1 than that of mice in the TPN group (All *P* < 0.05) (Figures 4A,B), as well as the levels in intestinal tissue.

### Effects of GLP-2 on Intestinal Inflammatory Cytokine Levels

**Figure 5:**
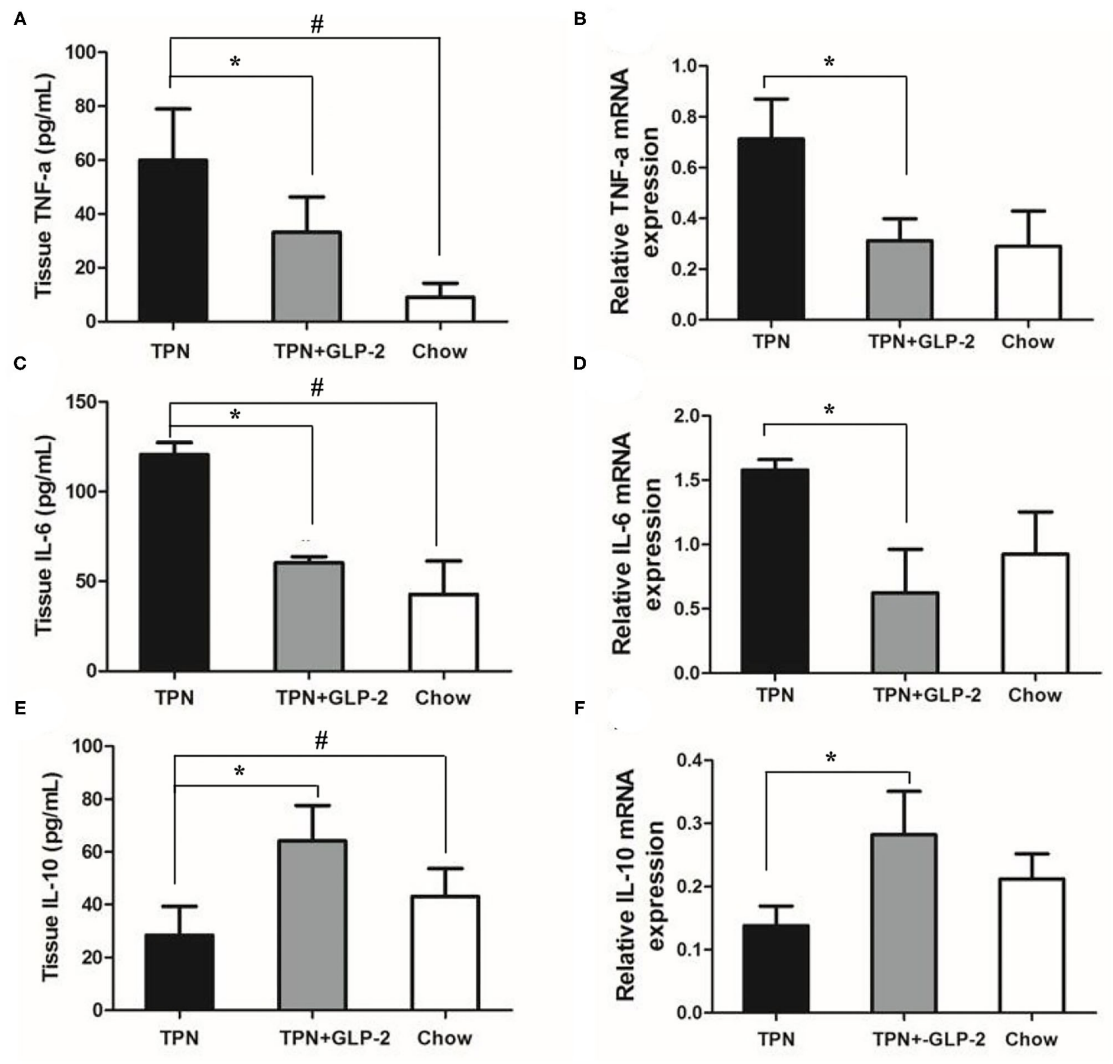
The intestinal inflammatory cytokine levels. **(A,B)** Expression of TNF-α protein and mRNA. **(C,D)** Expression of IL-6 protein and mRNA. **(E,F)** Expression of IL-10 protein and mRNA. Where ^*^indicates *p* < 0.05 for TNP vs. TPN+GLP-2 and Chow, and # indicates *p* < 0.05 for TNP vs. Chow.

Next, we tested the levels of inflammatory cytokine, such as pro-inflammatory cytokine TNF-α and IL-6, and anti-inflammatory factor IL-10. The TPN group had higher protein levels of TNF-α and IL-6, and lower levels of IL-10 than that of Chow group. Compared with the TPN groups, the proteins and mRNA levels of TNF-α and IL-6 were significantly decreased in the TPN+GLP-2 group (all *p* < 0.05), while the proteins and mRNA levels of IL-10 in the intestinal tissues were increased in the TPN+GLP-2 group (*p* < 0.05) (Figures 5A–F).

### Effects of GLP-2 on Serum D-lactic Acid and Endotoxin Levels and Inflammatory Infiltration of Intestinal Tissues

**Figure 6:**
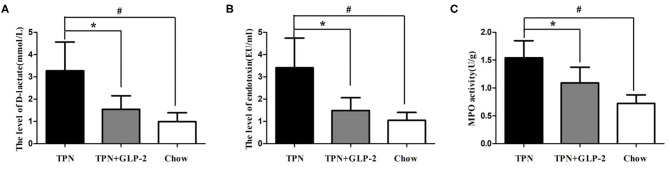
The plasma D-lactate and plasma endotoxin levels and inflammatory infiltration of intestinal tissues. **(A)** The plasma D-lactate level. **(B)** The plasma endotoxin level. **(C)** The intestinal tissue myeloperoxidase (MPO) activity. Where ^*^indicates *p* < 0.05 for TNP vs. TPN+GLP-2, and #indicates *p* < 0.05 for TNP vs. Chow.

Compared with the Chow group, the serum D-lactic acid and endotoxin levels were significantly increased in the TPN group (*p* < 0.05) (Figures 6A,B). And MPO activity in the intestinal tissues was also increased in the TPN group (*p* < 0.05) (Figure 6C). After GLP-2 treatment, the D-lactic acid and endotoxin levels, and MPO activity remarkably decreased.

## Discussion

In the case of intestinal function, patients are given priority to oral enteral nutrition. Nevertheless, it is undeniable that in many cases, there is no way for critically ill patients to achieve the goal of energy needs through oral enteral nutrition alone (3). At this time, supplementary parenteral nutrition or total parenteral nutrition is necessary. Parenteral nutrition has been a widely-used therapy for critically ill patients with gastrointestinal dysfunction, and has shown to improve their survival rates and prognosis and to promote rehabilitation outcomes (16). It is worth emphasizing that patients with intestinal integrity even if on PN, must restart minimal enteral feeding as soon as possible (17). The reason is that parenteral nutrition as a non-physiological method of nutrition delivery, has some negative effects on the gastrointestinal tract, including the intensification of intestinal mucosal atrophy, increased intestinal permeability and loss of intestinal integrity. Long term parenteral nutrition also inhibits immune function, which will lead to many clinical complications, such as increased bacterial translocation and sepsis (18, 19). Thanks to the normal human intestinal barrier structure (including mechanical, chemical, biological and immunological barriers), the rich bacterial pathogens in the intestinal tract cannot enter into human tissues (20). When the intestinal barrier is destroyed, endotoxin is likely to enter into the circulatory system and cause endotoxemia. Moreover, a significant decrease in the production of mucosa-derived growth factors has been reported in animal models of TPN (21). Therefore, for patients who rely on TPN, use the exogenous growth factors could be a hopeful approach to promote the growth of intestinal mucosa and improve epithelial barrier function (22). Many researches suggested that GLP-2 and its analogs play a significant role in patients with short bowel syndrome (SBS), including promoting intestinal adaptation, improving the surface area and absorption of the small intestine, and facilitating weaning from parenteral nutrition (23, 24). Therefore, it is of clinical significance to explore whether GLP-2 can improve TPN induced intestinal barrier injury by enhancing the intestinal immune function. Our study provides a possible method of patients who rely on IV fluid support to maintain longer intestinal barrier function. Indeed, 5 days of parenteral nutrition intervention is very short compared to treatment time in humans. However, previous studies suggested that the intestinal barrier damage has been severe after 5 days of PN intervention. Therefore, it is of certain significance to detect the influence of GLP-2 on intestinal function of mice after 5 days of during the process of IV infusion mainly consisted of nutritional materials (25, 26).

Intestinal innate immunity main consists of antimicrobial peptides produced and released by Paneth cells and mucin secreted by goblet cells (27). Paneth cells are mainly located at the bottom of the intestinal crypts near the stem cells and secrete a large amounts of antibacterial compounds, including lysozyme, sPLA2, RegIIIγ, Angiogenin4, and defensins (27, 28). These substances protect the epithelium by preventing bacterial adhesion. In the present study, TPN induced a significant down-regulation of the expression of lysozyme and sPLA2 in the small intestinal tissue, which is consistent with previous studies showing that PN can reduce Paneth cell function (29). Our study found that the application of GLP-2 reversed the expression of lysozyme and sPLA2 protein in mouse ileum tissue. These findings support our hypothesis that GLP-2 can improve the Paneth cells immune function during PN via IV infusion.

In this study, we found that the expression of epithelial protection and repair genes were significantly increased in the TPN+GLP-2 group. TPN can reduce the epithelial protection and repair protein synthesis, leading to intestinal barrier damage and bacterial translocation. Previous research suggested that PN combined with GLP-2 optimizes various intestinal parameters during post-resection adaptation compared to those observed with PN alone, including small intestine wet weight, villus and microvillus height, crypt cell proliferation, nutrient transporter expression, and nutrient absorption (30). Fgf7 promotes the proliferation of intestinal epithelial cells, which is also able to decrease the apoptosis induced by parenteral nutrition (31, 32). The administration of Fgf7 can reverse the current decline of anti-apoptotic Bcl-2 during TPN (30). The previous study found that the interactions of Fgf7 with the trefoil factor family promote intestinal mucosa repair (33). Our findings identify that GLP-2 could repair intestinal mucosal damage during PN via IV infusion.

In this study, the level of insulin like growth factor-1 protein and mRNA expression were significantly increased in the TPN+GLP-2 group. Previous studies have shown that IGF-1 can act on the intestinal epithelium through the endocrine effect of circulating IGF-1 mainly derived from hepatocytes and the paracrine effect of locally expression of IGF-1 synthesized by the intestinal mesenchyme (34). Growth hormone (GH), calorie and protein intake, and insulin have a positive regulatory effect on circulating IGF-1 circulation. Except in the case of extreme GH deficiency or excess, local intestinal IGF-1 is less regulated by GH but is positively regulated by luminal nutrients. There is some convincing evidence support that IGF-1 is an essential mediator of these effects on GLP-2, which stems from recent observations that GLP-2 cannot effectively increase the small intestine mass, crypt depth or villus height in mice with a targeted disruption of IGF-1 alleles (34) and mice with inducible deletion of IGF-1R specifically in intestinal epithelial cells (IECs) (35). Our research results are consistent with a previous study showing that GLP-2 enhances intestinal IGF-1 expression and also are required for small intestinal growth in response to GLP-2 (36). These findings confirm that IGF-1 is an important mediator of the intestinal tropic effect of GLP-2.

Numerous studies have documented damage to intestinal barrier function by pro-inflammatory cytokines in both intestinal epithelial and intestinal endothelial cells (37, 38). In contrast, some anti-inflammatory cytokines (such as: IL-10) have been shown to attenuate the intestinal epithelial barrier damage caused by IFN-γ. Therefore, it can be noticed that inflammatory cytokines can regulate the integrity of intestinal mucosal barrier. Previous studies found that TPN application reduced IL-4 and IL-10 levels in the intestine tissue, while increasing IFN-γ levels, which inhibited the production of IgA (39). In this study, we found that the proteins and mRNAs expression of TNF-α and IL-6 and MPO activity in the intestinal tissues were significantly increased in the TPN group, but IL-10 levels were decreased. GLP-2 could reverse the above changes in inflammatory factor expression levels.These results confirmed our hypothesis that GLP2 alleviates gut inflammation during on subjects whose nutritional needs are mainly rely upon IV infusion.

D-lactic acid is the metabolic end product of intestinal microorganisms such as Lactic Acid Bacteria and Escherichia coli (40). When the permeability of the intestinal mucosa increases, the D-lactic acid produced by the bacteria in the intestinal cavity enters the blood through the damaged mucous membrane, thereby increasing the plasma D-lactic acid concentration. Therefore, the D-lactic acid content in the peripheral blood can reflect the permeability of the intestinal mucosal barrier and the degree of impairment of intestinal mucosal barrier function. In this study, we also observed that both the D-lactic acid and serum endotoxin levels in the TPN group were higher than those in the TPN+GLP-2 group, which further demonstrated that GLP-2 could restored damage to the intestinal mucosal barrier during parenteral nutrition. Our results suggest that GLP-2 can alleviate gut inflammation and improve intestinal barrier function in line with previous studies (41, 42).

In conclusion, GLP-2 alleviated gut inflammation and improved enteric Paneth cells immune responses during PN via IV infusion in mice with a central catheter, possibly by improving the functioning of epithelial protection and repair and reducing mucosal inflammatory responses.

## Data Availability Statement

The raw data supporting the conclusions of this article will be made available by the authors, without undue reservation.

## Ethics Statement

The animal study was reviewed and approved by the animal Ethics Committee of Jinling Hospital (Ethical approval number: 2018NZGKJ-034).

## Author Contributions

XW developed the overall research plan and oversaw the study. GD and QL designed and conducted of the study, and completed the writing of the manuscript. XG, YZ, HZ, and JB helped the data collection and processing. The authors accept full responsibility for the design and conduct of the study, have access to the data, and controlled the decision to publish. All authors contributed to the article and approved the submitted version.

## Conflict of Interest

The authors declare that the research was conducted in the absence of any commercial or financial relationships that could be construed as a potential conflict of interest.

## Publisher's Note

All claims expressed in this article are solely those of the authors and do not necessarily represent those of their affiliated organizations, or those of the publisher, the editors and the reviewers. Any product that may be evaluated in this article, or claim that may be made by its manufacturer, is not guaranteed or endorsed by the publisher.

## Abbreviations

PN: parenteral nutrition
TPN: total parenteral nutrition
GLP-2: Glucagon-like peptide-2
ZO-1: zonula occludens-1
Tf1: trefoil factor-1
Tgf-b1: transforming growth factor-b1
Egfr: epidermal growth factor receptor
Hgf: hepatocyte growth factor
Fgf7: keratinocyte growth factor
Igf1: insulin like growth factor-1
sPLA2: secretory phospholipase A2 proteins
GH: Growth hormone.
