# Supplementary material for: Glucagon-Like Peptide-2 Modulates Enteric Paneth Cells Immune Response and Alleviates Gut Inflammation During Intravenous Fluid Infusion in Mice With a Central Catheter
Source: Front Nutr. 2021 Sep 3;8:688715. doi: 10.3389/fnut.2021.688715 (PMC8446534; doi:10.3389/fnut.2021.688715)
Supplement: Supplementary file 1 [file Table_1.doc]

**Table 1. The Primer Used for RT-qPCR Assays**

| **Gene** |  | **Sequence (5' – 3’)** |
| --- | --- | --- |
| **GAPDH** | Forward Primer | AGGCCGGTGCTGAGTATGTC |
|  | Reverse Primer | TGCCTGCTTCACCACCTTCT |
| **TNF-α** | Forward Primer | GGAACACGTCGTGGGATAATG |
|  | Reverse Primer | GGCAGACTTTGGATGCTTCTT |
| **IL-6** | Forward Primer | CCGCTATGAAGTTCCTCTCTGC |
|  | Reverse Primer | ATCCTCTGTGAAGTCTCCTCTCC |
| **IL-10** | Forward Primer | CCCATTCCTCGTCACGATCTC |
|  | Reverse Primer | TCAGACTGGTTTGGGATAGGTTT |
| **Tff1** | Forward Primer | AGCACAAGGTGATCTGTGTCC |
|  | Reverse Primer | GAAGCCACAATTTATCCTCTCCC |
| **Tff2** | Forward Primer | TGCTCTGGTAGAGGGCGAG |
|  | Reverse Primer | CGACGCTAGAGTCAAAGCAG |
| **Tff3** | Forward Primer | TTGCTGGGTCCTCTGGGATAG |
|  | Reverse Primer | TACACTGCTCCGATGTGACAG |
| **Tgfb1** | Forward Primer | CTTCAATACGTCAGACATTCGGG |
|  | Reverse Primer | GTAACGCCAGGAATTGTTGCTA |
| **Tgfb2** | Forward Primer | TCGACATGGATCAGTTTATGCG |
|  | Reverse Primer | CCCTGGTACTGTTGTAGATGGA |
| **Tgfb3** | Forward Primer | GGACTTCGGCCACATCAAGAA |
|  | Reverse Primer | TAGGGGACGTGGGTCATCAC |
| **Egfr** | Forward Primer | GCCATCTGGGCCAAAGATACC |
|  | Reverse Primer | GTCTTCGCATGAATAGGCCAAT |
| **Hgf** | Forward Primer | AACAGGGGCTTTACGTTCACT |
|  | Reverse Primer | CGTCCCTTTATAGCTGCCTCC |
| **Fgf7** | Forward Primer | TGGGCACTATATCTCTAGCTTGC |
|  | Reverse Primer | GGGTGCGACAGAACAGTCT |
| **Igf1** | Forward Primer | CCG AGG GGC TTT TAC TTC AAC AA |
|  | Reverse Primer | CGG AAG CAA CAC TCA TCC ACA A |
